# Challenges in identifying malnutrition in obesity; An overview of the state of the art and directions for future research

**DOI:** 10.1017/S095442242400012X

**Published:** 2024-04-05

**Authors:** Natasha Nalucha Mwala, Jos W. Borkent, Barbara S. van der Meij, Marian A.E. de van der Schueren

**Affiliations:** 1 Department of Nutrition, Dietetics and Lifestyle, School of Allied Health, HAN University of Applied Sciences, Nijmegen, the Netherlands; 2 Department of Human Nutrition and Health, Wageningen University and Research, Wageningen, the Netherlands; 3 Bond University Nutrition and Dietetics Research Group, Bond University, Gold Coast, Australia

**Keywords:** double burden, GLIM, malnutrition, obesity, sarcopenic obesity, screening tool

## Abstract

(Protein–energy) malnutrition in individuals living with obesity presents complex diagnostic challenges due to the distinctive physiological characteristics of obesity. This narrative review critically examines the identification of malnutrition within the population with obesity, distinguishing malnutrition in obesity from related conditions such as sarcopenic obesity. While noting some shared features, the review highlights key differences between these conditions. The review also highlights the limitations of current malnutrition screening tools, which are not designed for individuals living with obesity. These tools primarily rely on anthropometric measurements, neglecting (among others) nutrient intake assessment, which hinders accurate malnutrition detection. Additionally, this review discusses limitations in existing diagnostic criteria, including the Global Leadership Initiative on Malnutrition (GLIM) criteria, when applied to individuals living with obesity. Challenges include the identification of appropriate cut-off values for phenotypic criteria (unintentional weight loss, low body mass index and muscle mass) and aetiological criteria such as reduced food intake and inflammation for the population with obesity. Overall, this review emphasises the need for modified screening tools and diagnostic criteria to recognise and assess malnutrition in obesity, leading to improved clinical outcomes and overall wellbeing.

## Introduction

In recent years, there has been increasing recognition of the impact of disease-related malnutrition on both healthcare systems and patient outcomes^(1)^. Malnutrition is associated with higher hospital costs, prolonged length of stay, increased care burden, slower recovery and lower quality of life^(2–4)^. The prevalence of malnutrition varies among different populations, depending on the screening and diagnostic tools used, care setting, country and region, with estimates ranging from 10% to 50%^(3,5–7)^. Higher rates are often observed in specialised fields such as geriatrics, oncology and critical care units where patients are more vulnerable to nutritional deficiencies^(7–9)^. Thus, addressing malnutrition is important in preventing outcomes resulting from altered body composition, including unintentional weight loss and reduced muscle mass and function, greatly impacting overall wellbeing^(10,11)^.

Malnutrition is a broad term comprising several forms such as micronutrient deficiencies and protein–energy malnutrition (PEM), with conditions like overweight and obesity (hereinafter referred to as ‘obesity’) also being considered as a state of nutritional imbalance^(11,12)^. Despite these forms all having their causes and expressions, they share the underlying problem of an imbalance between energy and/or nutrient intake and requirements^(13,14)^. Most importantly, the presence of one form of malnutrition does not exclude the presence of another.

The presence of different types of malnutrition manifesting simultaneously is called the double burden of malnutrition (DBM)^(15,16)^. This can occur between different individuals in one living community, between different stages of life within one individual and for different forms of malnutrition that are present within one individual at the same time^(17)^. In this latter form, DBM mostly refers to individuals living with obesity who are suffering from micronutrient deficiencies in developing countries^(18,19)^. However, considering the nutrition-related problems in Western society, DBM comprises the coexistence of PEM and obesity^(20)^. Identifying PEM in individuals living with obesity becomes particularly challenging, as the excess weight associated with obesity can mask conventional indicators of PEM, such as involuntary weight loss and underweight.

In Western society, the identification of malnutrition has focused on undernutrition due to disease-related malnutrition and its consequences^(21)^. Thus, existing screening and assessment tools and the phenotypic criteria for malnutrition often rely on parameters such as unintentional weight loss, low body mass index (BMI) and reduced muscle mass^(22–25)^. The Global Leadership Initiative on Malnutrition (GLIM) criteria, for example, define malnutrition as the presence of at least one phenotypic criterion (unintentional weight loss, low BMI or reduced muscle mass) and at least one aetiologic criterion (reduced energy intake or disease burden/inflammation), indicating an imbalance between energy and/or nutritional intake and requirements with implications for body composition, function and clinical outcomes^(26)^.

However, these parameters may not effectively capture malnutrition in individuals living with obesity. For instance, underweight (defined as BMI <18·5 kg/m^2^) is unsuitable for identifying malnutrition in this group^(27–29)^. Furthermore, traditional cut-off values for unintentional weight loss and reduced muscle mass might not be applicable, given the distinct body composition of individuals living with obesity in comparison with those with a normal BMI^(30)^. Similarly, aetiological criteria such as decreased appetite and reduced food intake might require adjusted cut-off values for individuals living with obesity.

To warrant an early identification and improve treatment opportunities, more knowledge is needed on the specific problem of malnutrition in obesity. Therefore, the objective of this narrative review is to delve into the identification of malnutrition in individuals living with obesity. This review aims to distinguish malnutrition among individuals living with obesity from the closely related clinical condition of sarcopenic obesity, discuss the difficulties in diagnosing malnutrition in this context, evaluate the limitations of current malnutrition screening tools and assessment criteria, and propose recommendations for further research. By addressing these key aspects, this review seeks to deepen the understanding of malnutrition in individuals living with obesity. It emphasises the importance of implementing more effective screening and assessment approaches to identify and support individuals living with obesity at risk of malnutrition.

### The difference between sarcopenic obesity and malnutrition in obesity

Sarcopenic obesity and malnutrition in obesity share some similarities but also exhibit distinct characteristics. Sarcopenic obesity is defined by the coexistence of obesity (high fat mass) and sarcopenia (low muscle functionality and altered body composition)^(31–34)^. In contrast, malnutrition in obesity refers to undernutrition related to specific energy or nutrient deficiencies (such as PEM) within the context of obesity^(11,34)^.

**Fig. 1:**
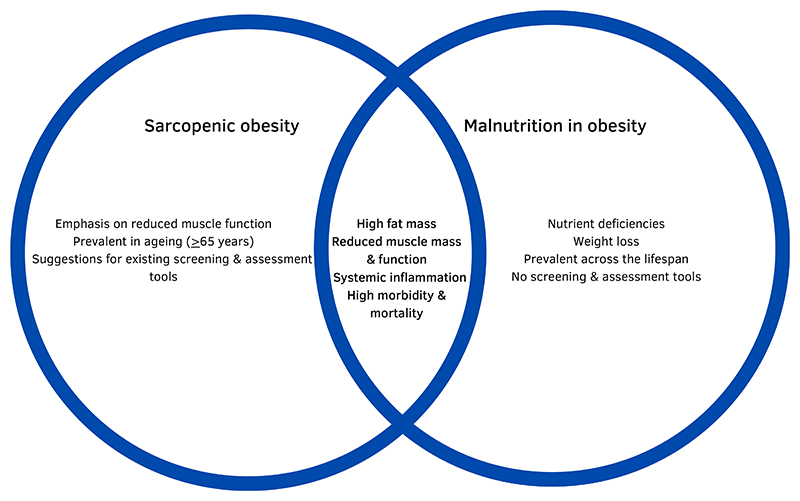
Differences and similarities of sarcopenic obesity and malnutrition in obesity.

Despite common factors like high fat mass, reduced muscle mass, systemic inflammation and consequences such as a higher risk of morbidity and mortality^(33)^, recognising the differences between the two conditions is important. Malnutrition in obesity encompasses a broader range of nutritional deficiencies, including unintentionally reduced energy and/or nutrient intake leading to weight loss, setting it apart from sarcopenic obesity^(12)^. Sarcopenic obesity manifests in two forms: age-related (primary) and disease-related (secondary). Both involve a decline in muscle function and altered body composition, which may not necessarily be attributed to nutritional deficiencies^(35,36)^. Figure 1 illustrates the key characteristics of sarcopenic obesity and malnutrition in obesity, effectively highlighting their differences and similarities.

A notable difference between these conditions lies in their prevalence across different age groups. Although sarcopenic obesity, particularly the secondary form, associated with specific comorbidities such as cancer or rheumatoid arthritis, can occur in individuals under 65 years, the primary form is more prevalent and particularly observed in those aged 65 and older^(35,37,38)^. Sarcopenic obesity in the elderly is mainly attributed to age-related hormonal changes that can lead to a decrease in hormones critical for maintaining muscle mass, such as insulin-like growth factor-1 (IGF-1), testosterone, dehydroepiandrosterone (DHEA) and oestrogen^(35,39)^. In contrast, malnutrition in obesity can occur across the lifespan, affecting individuals of different ages^(15,17,40)^.

Another difference between sarcopenic obesity and malnutrition in obesity is the focus on muscle function. While both conditions commonly exhibit reduced muscle mass and function, the algorithm for defining sarcopenic obesity prioritises muscle function over muscle mass^(10,32)^. Additionally, impaired physical performance is integral to the definition^(33)^. Diagnostic procedures for sarcopenic obesity begin with an assessment of skeletal muscle function, followed by an assessment of body composition. The confirmation of the diagnosis involves identifying the presence of excess adiposity and low skeletal muscle mass or related body compartments^(39,41)^.

Furthermore, a difference emerges in the screening and assessment approaches for these two conditions. For sarcopenic obesity, a diagnostic procedure has already been proposed. This starts with screening by evaluating high fat mass through measures such as BMI and waist circumference, along with surrogate markers for sarcopenia. The subsequent diagnosis then includes an analysis of muscle function and body composition^(31)^. In contrast, there are no specific screening or assessment tools available for identifying malnutrition within obesity. Despite the existence of screening and assessment methods for sarcopenic obesity, a notable similarity arises: the absence of specific cut-offs for both conditions. The Sarcopenic Obesity Global Initiative (SOGLI) expert panel has recognised the lack of specific cut-offs for sarcopenic obesity and formulated research questions to further define muscle function and body composition measures for sarcopenic obesity^(32,42)^. Meanwhile, the group supports the use of BMI and waist circumference cut-offs while recognising their limitations. Similarly, cut-offs for muscle function and muscle mass have been suggested, with a recommendation to adopt them from previous studies^(43–47)^. This highlights the need for future research to define optimal cut-off points for application in both research and clinical practice for sarcopenic obesity, as well as for screening and assessing malnutrition in overweight and obesity, where defined cut-off points are currently non-existent.

### The challenge with diagnosing malnutrition in obesity

Despite the widespread use of the GLIM criteria for diagnosing malnutrition, challenges arise when applying these criteria to patients with obesity. The phenotypic criterion of low BMI is not useful in individuals living with obesity^(28)^. Moreover, the suggested cut-off values for recent weight loss and reduced muscle mass in the GLIM guidelines may also not be applicable for individuals living with obesity as these cut-off points are established in studies with individuals with a normal BMI^(26,48^).

Weight loss can be defined and reported using different measures, including percentage weight loss, changes in weight in kilograms and changes in BMI (kg/m^2^)^(49)^. In the GLIM criteria, unintentional weight loss is specifically defined and reported as a percentage of an individual’s usual weight^(26)^. This choice can lead to major differences when interpreting weight loss outcomes, especially in the context of obesity, with a pronounced impact on individuals living with obesity. To illustrate this, let us consider two individuals with different body weights. A weight loss of 3 kg may greatly impact an individual weighing 60 kg, representing a 5% reduction in body weight, which is considered clinically relevant if assumed over 3 months^(50)^. However, in an individual weighing 160 kg, the same 3 kg weight loss would represent only a 1·9% reduction in body weight, which may not be as clinically relevant. This discrepancy highlights how using percentages for weight loss in individuals living with obesity may require a considerable number of kilograms lost before reaching the threshold for malnutrition diagnosis, making it challenging to accurately assess malnutrition risk in this population.

In individuals living with obesity, involuntary weight loss impacts all-cause mortality, but the severity is less pronounced than in individuals with normal weight and underweight^(51)^. Research indicates that ±5% involuntary weight loss in a population with obesity, unlike that in a population with a normal BMI, is not associated with increased mortality, and higher weight loss percentages might be more indicative of malnutrition risk^(52)^. NHANES data reveal that weight loss beyond 15% increases mortality risk in individuals living with obesity, with no significant association for losses between 5% and −15%, except among women who are overweight^(53)^. Hospitalised patients’ data (from the nutritionDay data collection) reinforces this, showing that mortality risk increases with weight loss across all BMI categories, but in the BMI category above 30 kg/m^2^, this becomes significant only when the loss exceeds 12·6%^(54)^. Additionally, distinguishing between fat and muscle mass loss during weight loss is important, given the increased risk of conditions like sarcopenia associated with muscle mass loss^(55)^. Furthermore, malnutrition diagnostic criteria that investigate unintentional weight loss may also face challenges in assessing malnutrition in patients with obesity. Due to the desire for weight loss among many individuals living with obesity, they may engage in unhealthy weight loss practices and not report weight loss as unintentional, possibly leading to an increased malnutrition risk that goes unnoticed^(28,56)^.

Assessing muscle mass is vital in nutritional assessment for individuals living with obesity but presents specific challenges. Studies suggest that a higher BMI is associated with a higher muscle mass due to the additional weight, comprising both fat and muscle^(55,57)^. This association can lead to an overestimation of muscle mass when using methods like upper-arm or calf circumference, as excess fat can distort measurements, potentially underdiagnosing malnutrition^(58)^. To address this, there have been proposals for age and BMI-specific cut-off points for calf circumference^(59)^. Furthermore, techniques such as dual-energy X-ray absorptiometry (DEXA) and computed tomography (CT) scans have a weight limitation, that is, the maximum body weight that the equipment is designed to accommodate while still providing accurate results. This limitation can pose difficulties in obtaining precise measurements in individuals living with obesity. Bio-electric impedance (BIA) measurements are less reliable in this population due to the overestimation of fat-free mass (FFM) by BIA equations, probably explained by a higher hydration of FFM in adipose tissue^(60)^. Conversely, ultrasonography shows potential as a non-invasive and accessible technique for accurately evaluating muscle mass and its characteristics in individuals living with obesity. However, challenges such as a lack of standardisation need to be addressed for its full integration into clinical practice^(61)^.

Additionally, these techniques can also misclassify reduced muscle mass within obesity, where the difference between muscle mass in absolute terms (kg) and relative terms (%) is high^(58,62–64)^. Since cut-off values are based on absolute terms, individuals living with obesity may have a relatively low muscle–fat ratio but still possess a relatively high muscle amount in kg, especially with high adipose tissue. Thus, many individuals could exceed the cut-off for reduced muscle mass, potentially escaping malnutrition diagnosis^(25)^.

Applying the GLIM aetiologic criterion of low food intake (a 50% reduced food intake cut-off) in individuals living with obesity presents challenges^(26)^. Bariatric surgery, especially gastric sleeve surgery, has gained popularity as an alternative treatment for obesity, particularly in those with morbid obesity (BMI >40 kg/m^2^)^(65)^. However, this surgery has implications for the dietary intake of individuals with obesity, resulting in a decrease in nutrient intake and absorption, potentially compromising accurate screening of the GLIM criterion related to food intake^(66)^. Moreover, this criterion may not accurately reflect inadequate nutrient intake in individuals living with obesity due to their higher nutritional requirements based on their higher body weight^(18)^. Based on literature, the most reliable energy intake data indicate high levels, exceeding 4000 kcal/d (16,736 kJ/d) for individuals maintaining weight stability at the highest levels of morbid obesity^(67)^. Thus, a 50% intake reduction in this population remains a substantial intake. Furthermore, determining the appropriate cut-off values for reduced food intake in individuals living with obesity accustomed to overeating is challenging. This is due to their different eating patterns and nutritional requirements compared with those with healthy body weights^(68)^.

Inflammation triggers increased protein turnover, which leads to the loss of muscle mass, strength and function^(69)^. For this reason, inflammation has been incorporated into the aetiologic criteria of the GLIM definition^(26)^. Determining suitable thresholds for disease burden and inflammation in individuals living with obesity remains uncertain. Obesity often involves a mild, chronic inflammation driven by adipose tissue releasing pro-inflammatory cytokines^(70)^, consequently increasing C-reactive protein (CRP) levels^(71)^. An individual meets the GLIM aetiologic criterion of inflammation if they show repeated CRP levels above 3·0 mg/L^(72)^. In individuals with metabolic syndrome, obesity primarily drives CRP elevation, typically exceeding 1·0 mg/L, indicating inflammation^(73)^. Thus, many individuals living with obesity would meet the GLIM criteria’s inflammation aetiologic criterion, potentially resulting in false positives due to the prevalent low-grade inflammation in obesity^(74)^. The applicability of the provided guidance for the GLIM criteria’s inflammation aetiologic criterion may vary within the population with obesity due to the variability of obesity-related inflammation, influenced by factors such as obesity duration, genetic predisposition and underlying health conditions^(13)^.

Moreover, the coronavirus disease 2019 (COVID-19) pandemic highlighted major differences in disease outcomes as a result of systemic inflammation stemming from pre-existing conditions in patients such as diabetes and obesity^(75)^. The guidance for the GLIM criteria’s inflammation aetiologic criterion outlines a categorised list of example diseases based on inflammation levels ranging from mild to severe^(72)^. The risks of these diseases might be different between individuals living with obesity and those who do not live with obesity, with individuals living with obesity carrying higher risks of severe consequences^(76)^.

Obesity can alter disease experiences, potentially worsening disease severity due to factors such as compromised immune function, increased disease risk, additional stress on the cardiovascular and respiratory systems and limited treatment effectiveness^(77,78)^. This can result from altered pharmacokinetics and potential side effects of disease treatments, such as chemotherapy^(79,80)^. Consequently, managing chronic conditions becomes more challenging as surgical risks increase and certain diseases such as congestive heart failure, diabetes and rheumatoid arthritis may progress faster in the presence of obesity^(81)^.

In summary, both the phenotypical and aetiological aspects of the widely accepted GLIM criteria for diagnosing malnutrition may not fully address the complexities of diagnosing malnutrition in the context of individuals living with obesity as the cut-off and threshold values used are not specifically tailored for the population with obesity. Further research and adaptations of the criteria should aim at integrating adapted cut-off values within the GLIM criteria to also suit the specific nutritional challenges faced by individuals living with obesity, similar to the approach taken with ethnicity-specific cut-offs. The current reference values in the GLIM criteria may not be entirely applicable to this population, warranting further investigation and modification to accurately identify and diagnose malnutrition in individuals living with obesity.

### The challenge with current malnutrition screening tools

The foundation of malnutrition diagnosis according to the GLIM criteria relies on the use of a validated screening tool^(26)^. Various screening tools have been developed and validated to assess malnutrition in different populations and healthcare settings^(82–84)^. Table 1 provides an overview of frequently used and validated malnutrition screening tools^(85)^. These tools encompass a range of criteria, incorporating phenotypic aspects such as unintentional weight loss, low BMI and reduced muscle mass, alongside aetiologic indicators such as reduced food/fluid intake, disease burden/inflammation and risk factors for malnutrition^(26,85,86)^.

Most malnutrition screening tools, following the 2002 European Society for Clinical Nutrition and Metabolism (ESPEN) guidelines, primarily rely on low BMI and recent (unintentional) weight loss to identify malnutrition risk^(48,87)^. As previously mentioned, a low BMI (<18·5 kg/m^2^) is not useful, and traditional cut-off values for unintentional weight loss might not apply to individuals living with obesity, despite inclusion in these screening tools. In addition, because of the emphasis on weight loss, healthcare providers might not consider screening individuals living with obesity for malnutrition risk^(28)^. In widely used malnutrition screening tools such as the MNA-short form (MNA-SF) and MUST, there are distinct differences in BMI cut-off points^(88)^. The MNA-SF sets a cut-off at 23 kg/m^2(89)^, whereas the MUST adopts a different approach with a cut-off of 20 kg/m^2(90)^. Nevertheless, both tools may not be suitable for individuals living with obesity, and their underlying scoring system might therefore not apply to this specific population. Thus, a pertinent question arises of whether the traditional malnutrition screening tools are still appropriate^(85)^.

Moreover, it is crucial to note that most malnutrition screening tools were validated against ‘gold standards’^(85,87)^, primarily focused on phenotypical criteria such as unintentional weight loss, low BMI and reduced muscle mass. Thus, many screening tools lack aetiological criteria. In addition, the use of validated tools specific to the setting and population is of utmost importance^(91)^. Within the context of obesity, only one screening tool has been proposed for assessing malnutrition: the JaNuS tool^(92)^. This tool screens for over- and undernutrition in two separate sections, enabling a patient to score positively for one or both conditions. However, the JaNuS tool was validated in a pre-geriatric population and uses low albumin and low lymphocyte count as part of its criteria to assess nutrition status, which poses limitations to its universal applicability^(92,93)^. This further emphasises the urgent need to develop and validate specific tools designed to accurately identify malnutrition in this context.

In many malnutrition screening tools, assessing reduced muscle mass is often overlooked, except in the MNA-SF and SNAQ^65^, which use calf and arm circumferences^(85,86)^. Again, the traditional cut-off values for these measurements may not apply to individuals living with obesity^(94)^. Due to higher fat mass presence, muscle mass and predominantly fat mass will be included in the measurement, rendering it inaccurate^(58)^. Thus, to effectively address this challenge, questions need to be adapted to include appropriate cut-offs specifically tailored to the context of obesity.

Malnutrition screening tools typically strongly rely on anthropometric measurements and focus only to a lesser extent on nutrient intake^(41)^. Most screening tools lack comprehensive questions regarding nutritional intake, let alone their applicability to obesity^(88)^. As a result, these tools often fail to effectively identify the underlying nutritional issues. Notably, questions related to nutrient intake in malnutrition screening tools tend to focus solely on quantity, without considering the distinction between the quality and quantity of nutrient consumption. This oversight is particularly important given that obesity is influenced by the quality of foods consumed rather than just their quantity^(95,96)^. Furthermore, screening tools often fail to consider an individual’s specific dietary preferences and restrictions, which could lead to incomplete dietary pattern assessments due to reliance on binary yes-or-no responses^(83,88)^. This limitation might undermine the accurate evaluation of nutritional risks.

Besides the phenotypic and aetiological criteria, malnutrition screening tools take various risk factors into account, as shown in Table 1^(85)^. However, not all these factors apply to obesity. For instance, appetite regulation differs in individuals living with obesity due to disrupted mechanisms of appetite control and body weight maintenance, leading to distinct changes in appetite and dietary intake responses^(97)^. Satiety may also not be a suitable malnutrition risk factor for individuals living with obesity given the hormonal imbalances and metabolic dysregulation often present in obesity, potentially resulting in reduced satiety^(98)^. This contrasts with cases of malnutrition where reduced appetite and satiety are more common. Hence, the importance of satiety as a malnutrition risk factor could vary between individuals living with obesity and those with a healthy weight.

Certain screening tools, including the MNA and SCREEN II, extend the standard weight loss question to incorporate additional obesity-related factors such as intentions for weight change and perceptions of body weight^(86)^. However, relying on self-perception of nutritional status in individuals living with obesity could be compromised by societal norms and personal biases, potentially leading to an underestimation of malnutrition risk^(99)^. This challenge highlights the need for more objective measures in nutritional screening. Furthermore, tools designed to assess self-perception can also be influenced by the desire for weight loss, possibly underestimating nutritional sufficiency and leading to a higher nutritional risk^(100)^. Hence, relying solely on self-reported perceptions, especially in cases of obesity, may not ensure accurate assessments.

There is also a noteworthy gap in screening tools regarding malnutrition risk factors associated with obesity. For example, these screening tools often omit recurrent cycles of weight loss and gain resulting from yo-yo dieting and unhealthy weight loss practices, which can reveal underlying nutritional challenges^(101)^. These cycles should not be ignored, as they pose potential risks including metabolic adaptations, loss of lean muscle mass, psychological consequences, cardiovascular health concerns and the potential for disordered eating^(102)^. Screening an individual living with obesity’s treatment history is equally crucial but not incorporated into screening tools. Consider the already mentioned bariatric surgery, for instance. It is important due to its association with reduced protein intake resulting from post-surgical dietary restrictions and intolerance to protein-rich foods, thus acting as a risk factor for malnutrition^(66)^.

In conclusion, current malnutrition screening tools may not accurately identify malnutrition in the population with obesity, a concern heightened by the rising global obesity rates. Our examination of this issue has revealed several knowledge gaps that need to be filled.

### Recommendations for future research, within our and other projects

As we look ahead, our research efforts aim to centre around the following recommendations. These suggestions stem from our insights into challenges such as differentiating between sarcopenic obesity and malnutrition in obesity, improving diagnostic accuracy and updating screening tools for identifying malnutrition within obesity. By adopting these recommendations, we can facilitate more precise identification, interventions and care for individuals with both obesity and malnutrition.

#### Differentiating sarcopenic obesity from malnutrition in obesity

Research efforts should be directed towards refining the differentiation between sarcopenic obesity and malnutrition among individuals living with obesity. The focus should centre on understanding their distinct pathogenesis linked to inflammatory patterns and severity. This should involve the investigation of specific (inflammatory) biomarkers, imaging techniques or composite indicators that can accurately distinguish between these conditions. Additionally, it is important to consider the association of both conditions with the ageing process (sarcopenic obesity) and acute diseases (malnutrition in obesity). These efforts will ensure appropriate intervention strategies for optimal health outcomes.

#### Improving diagnostic accuracy

Novel diagnostic approaches that consider the altered physiology of obesity should be developed and validated within the population with obesity. This involves improving anthropometric measurements, body composition analyses and exploring biochemical markers. This will facilitate accurate identification and diagnosis of the problem and timely intervention.

#### Updating screening tools and diagnostic criteria

Recognising the limitations of current screening tools and diagnostic criteria, future research should focus on the development and validation of comprehensive assessment tools and diagnostic criteria tailored to the complexities of obesity. These tools should incorporate refined body composition assessments, inflammation criteria and applicable obesity-related risk factors that accurately reflect the nutritional status of individuals living with obesity. Furthermore, these tools should incorporate distinct cut-off points tailored for weight loss and muscle mass in individuals living with obesity. A constructive proposal would be to integrate these modifications within the GLIM criteria, forming a dedicated subset specially designed for patients with obesity.

#### Validation across diverse populations with obesity

To ensure the applicability of newly developed screening tools and diagnostic criteria, validation studies should be conducted across diverse populations with obesity, encompassing different age groups, ethnicities and specific comorbidities. This will enhance their usability across different demographic and clinical situations, making them more effective in real-world settings.

#### Conducting longitudinal studies for comprehensive insights

Conducting longitudinal studies that follow the nutritional trajectory of individuals living with obesity as well as those with a healthy weight over time is essential for meaningful comparisons. These studies can offer valuable insights into the interplay between obesity, malnutrition and disease progression, aiding in identifying early markers of malnutrition (risk) and the evaluation of interventions.

#### Collaborative research endeavours

Promoting collaboration among experts in malnutrition, obesity and related fields is crucial for collectively addressing the complex challenges of malnutrition assessment in individuals living with obesity. This collaborative approach can drive comprehensive research strategies, leading to the development of more accurate and impactful assessment tools. Our existing partnership with SOGLI, which specialises in sarcopenic obesity research, provides a unique advantage and can further enhance these efforts^(31,32)^. Additionally, we also plan to collaborate with the recently established Global Leadership Initiative on Sarcopenia (GLIS), closely following its outcomes to contribute to and stay informed about advancements in sarcopenia research. By using shared knowledge and resources, we can accelerate the innovation of assessment tools, interventions and guidelines tailored to identifying and addressing malnutrition within the context of obesity.

#### Interventional trials for optimal care

Research can be broadened through the implementation of interventional trials that prioritise tailored strategies to address malnutrition in individuals living with obesity. These trials can provide an approach to explore the effects of personalised nutritional interventions, exercise plans and multidisciplinary care approaches on augmenting nutritional wellbeing and overall health outcomes. By systematically addressing these research recommendations, the scientific community can bridge the current gaps in malnutrition assessment within the context of obesity, leading to advancements in diagnosis, intervention and, ultimately, improved health outcomes for individuals living with obesity.

## Conclusion

Addressing malnutrition within the context of obesity is a multidimensional challenge. The key points from this review reveal that traditional malnutrition diagnostic criteria are unsuitable for individuals living with obesity due to the distinctive physiological characteristics of obesity. While the GLIM criteria are widely recognised, they require distinct cut-off points for individuals living with obesity. The usage of certain indicators such as low BMI and percentage weight loss could potentially lead to a malnutrition underdiagnosis. Similarly, existing screening tools may also fall short in capturing the nutritional challenges faced by individuals living with obesity, as they prioritise anthropometric measurements over specific nutrient intake considerations.

This overview emphasises the urgent need for tailored approaches that acknowledge the details of malnutrition in the context of obesity. The call for adapting existing tools is evident, requiring the integration of appropriate cut-off values for weight loss and reduced muscle mass that are specific for obesity. By doing so, healthcare practitioners will be better equipped to identify and address malnutrition in individuals living with obesity at an early stage, ultimately leading to improved healthcare outcomes and overall wellbeing.

## Figures and Tables

**Table 1:** Overview of criteria in a selection of frequently used malnutrition screening tools (from de van der Schueren, 2022)

	Phenotypic criteria				Aetiologic criteria				Risk factors							
Screening tool	Weight change	Low BMI	Body composition		Food/fluid intake	GI symptoms	Disease burden/inflammation (including inflammation parameters)		Loss of appetite	Higher age	Mobility/ activity	Functional capacity/ muscle function	Feeling full		Altered taste/ smell	Inability to eat/needing help with eating
**DETERMINE** ^1^	x				x		x										Identifying malnutrition in obesity
**GNRI** ^2^	x					x	x		x								
**MNA-SF** ^3^	x	x	x		x	x	x		x		x						
**MST** ^4^	x				x				x								
**MUST** ^5^	x	x			x		x										
**NRI** ^6^	x	x					x		x							x	
**NRS** ^7^	x	x			x	x	x		x							x	
**NRS 2002** ^8^	x	x			x		x			x							
**NUFFE** ^9^	x				x	x	x		x		x					x	
**PG-SGA SF** ^10^	x				x	x			x		x		x		x		
**SCREEN II** ^11^	x				x				x								
**SNAQ** ^12^	x								x								
**SNAQ^RC,^** ^13^	x	x							x								
**SNAQ^65^** ^+,14^	x		x						x			x				x	
**SNAQ** ^15^									x				x		x		
	Risk factors														Other		
Screening tool	Problems with buying or preparing food (including finances)	Self-perception of nutritional status	Decreased/ inadequate intake		Mouth problems/ problems biting, chewing, swallowing, coughing	Eating alone/ company at meals	Alcohol intake		Drugs intake	Neuropsychological problems	Health state	Pain	Fatigue		Use of sip feeding or tube feeding		
**DETERMINE** ^1^	x		x		x	x	x		x								
**GNRI** ^2^																	
**MNA-SF** ^3^		x			x					x							
**MST** ^4^																	
**MUST** ^5^			x														
**NRI** ^6^																	
**NRS** ^7^					x												
**NRS 2002** ^8^			x														
**NUFFE** ^9^	x					x			x		x						
**PG-SGASF** ^10^	x		x		x							x	x		x		
**SCREEN II** ^11^	x	x	x		x	x											
**SNA**Q^12^															x		
**SNAQ^RC,^** ^13^																	
**SNAQ^65^** ^+,14^																	
**SNAQ** ^15^																	

1 DETERMINE Your Nutritional Health Nutrition Screening Initiative.

2 Geriatric Nutrition Risk Index.

3 Mini Nutritional Assessment Short Form.

4 Malnutrition Screening Tool.

5 Malnutrition Universal Screening Tool.

6 Nutritional Risk Index.

7 Nutrition Risk Score.

8 Nutritional Risk Screening 2002.

9 Nutritional Form for the Elderly.

10 Patient-Generated Subjective Global Assessment Short Form.

11 Seniors in the Community: Risk Evaluation for Eating and Nutrition, version II.

12 Short Nutritional Assessment Questionnaire.

13 Short Nutritional Assessment Questionnaire for the Residential Care.

14 Short Nutritional Assessment Questionnaire 65Þ.

15 Simplified Nutritional Appetite Questionnaire.
